# Extensive Epidural Hemorrhage Associated with Thoracolumbar Disc Extrusion in French Bulldogs

**DOI:** 10.3390/vetsci11110573

**Published:** 2024-11-16

**Authors:** Julius Underberg, Arianna Maiolini, Maja Waschk, Daniela Schweizer

**Affiliations:** 1 Division of Clinical Radiology, Department of Clinical Veterinary Science, Vetsuisse Faculty, University of Bern, 3012 Bern, Switzerland; maja.waschk@unibe.ch (M.W.); daniela.schweizer@unibe.ch (D.S.); 2 Division of Clinical Neurology, Department of Clinical Veterinary Science, Vetsuisse Faculty, University of Bern, 3012 Bern, Switzerland; arianna.maiolini@unibe.ch

**Keywords:** intervertebral disc extrusion, epidural hemorrhage, dorsal epidural space, intramedullary bleeding

## Abstract

(1) Background: Intervertebral disc extrusion may be accompanied by extensive epidural hemorrhage (EEH) and result in spinal cord compression. EEH is more commonly seen in French Bulldogs compared to other breeds. The majority of French Bulldogs have lumbar intervertebral disc extrusion, but it is unclear if there is an association between the site of thoracolumbar disc extrusion and EEH. This retrospective study’s aim was to investigate the association and prevalence between the site of thoracolumbar intervertebral disc extrusion and EEH in French Bulldogs. (2) Methods: Thoracolumbar MRI of French Bulldogs was examined for the site of intervertebral disc extrusion and the presence, distribution, and extent of EEH. (3) Results: In 148 French Bulldogs with thoracolumbar intervertebral disc extrusion, the most common location was L4/L5 (22%), followed by L3/L4 (20%) and T13/L1 (15%), and 70.3% had EEH. The highest incidence of EEH across all thoracolumbar sites was seen at L1/L2 (84.2%), followed by L5/L6 (81.3%) and L2/L3 (72.2%). The incidence of EEH was 70.5% across all lumbar sites and 66.9% for all thoracic sites. (4) Conclusion: In this population of French Bulldogs, the prevalence of lumbar disc extrusion was high, and 70.3% had EEH. There was no significant difference between the site of thoracolumbar intervertebral disc extrusion and EEH.

## Simple Summary

French Bulldogs often suffer from disc herniations leading to paralysis of the hind limbs. In this breed especially, a high number of dogs show associated bleeding in the vertebral canal that, additionally to the disc herniation, can lead to compression of the spinal cord. We analyzed magnetic resonance images of 148 French Bulldogs to see if bleeding occurred more often at certain locations of disc herniation. We found that the most common location of disc herniation in French Bulldogs was L4/L5, which is different compared to other breeds, and that in 70% of these dogs, disc herniation was accompanied by extensive bleeding. The highest percentage of disc herniations with extensive bleeding was seen with herniation between the first and second lumbar vertebra.

## 1. Introduction

Intervertebral disc extrusion can result in the traumatic rupture of the ventral venous plexus, leading to epidural hemorrhage. The extent of epidural hemorrhage is variable and may span multiple spinal cord segments, potentially necessitating decompressive surgery at more than one site [1]. This phenomenon is described as disc extrusion with extensive epidural hemorrhage, without further specifying the length and compression of the epidural hemorrhage [2]. Epidural hemorrhage is characterized by the presence of erythrocytes and macrophages containing blood degradation products, which are believed to trigger epidural inflammation. Epidural inflammation is important to consider in cases of intervertebral disc herniation, as paraplegic medium- to large-breed dogs with extensive epidural hemorrhage have less favorable outcomes [3]. In this context, it is important to emphasize that the contact area between the epidural hemorrhage and the spinal cord seems to be more critical for prognosis than the extent of spinal cord compression, and dogs with minimal epidural inflammation have a better prognosis for regaining ambulation [4]. Domestic dog breeds with the chondrodystrophic phenotype are strongly associated with the expression of the FGF4 retrogene (fibroblast growth factor 4) [5]. Specifically, the FGF4 retrogene on chromosome 12 appears to be associated with intervertebral disc disease [6].

French Bulldogs appear to develop disc-associated epidural hemorrhage more frequently than Dachshunds, other chondrodystrophic breeds, and non-chondrodystrophic dogs [7,8].

Across all dog breeds, the most common site for intervertebral disc extrusion is at T12/T13, but epidural hemorrhage seems significantly more common in intervertebral disc extrusion at L4/5 and less common at the thoracolumbar junction [9]. However, the association between the location of intervertebral disc herniation and epidural hemorrhage is debated and another study found that in French Bulldogs, epidural hemorrhage is associated with caudal thoracic and thoracolumbar intervertebral disc extrusion [8]. Based on our observations, French Bulldogs at our institution frequently present with epidural hemorrhage in cases of lumbar intervertebral disc extrusion.

The aim of this retrospective study was to determine the prevalence of extensive epidural hemorrhage (EEH) at specific sites of intervertebral disc extrusion in the thoracic and lumbar spine in a population of French Bulldogs and whether certain sites are at an increased risk of developing EEH. We hypothesized that EEH has a higher prevalence with intervertebral disc extrusion at specific caudal lumbar sites.

## 2. Materials and Methods

Inclusion criteria were French Bulldogs that presented with paraparesis or paraplegia localized to T3–L3 or L4–S3 spinal cord segments, with a clinical onset of less than 14 days and an MRI diagnosis of intervertebral disc extrusion in a 10-year time frame from 2012–2022. Dogs were classified according to the presence or absence of deep pain perception. The deep pain positive (DPP) group included dogs showing different degrees of paraparesis and paraplegia with preserved deep pain. The deep pain negative (DPN) group included paraplegic dogs without deep pain perception. Dogs showing unilateral sensorimotor loss were grouped into the DPP group. Further inclusion criteria were an MRI examination protocol including a T2-weighted sequence in the sagittal and transverse plane, a T1-weighted sequence in the transverse or dorsal plane, a T2-weighted fat-suppressed sequence in the dorsal plane, and a T2* gradient echo sequence in the transverse plane. Exclusion criteria were dogs where MRI images did not allow for correct counting of the vertebrae or incomplete sequence acquisition. The vertebra with the last pair of ribs was defined as T13.

The images of this single institution study were acquired with a 1.0 T magnet (Philips HFO Panorama, Philips Medical Systems, PC Best, The Netherlands), a 1.5 T magnet (Avanto, Siemens Healthineers, Erlangen, Germany), or a 3.0 T magnet (Magnetom Vida 3T, Siemens Healthineers, Erlangen, Germany).

Images were evaluated by a senior resident (JU) and a board-certified radiologist (DS) in consensus, blinded to the original report, using a DICOM viewer (DeepUnity Viewer, Dedalus Healthcare GmbH, Bonn, Germany). Images were assessed for the location of intervertebral disc extrusion based on narrowing of the intervertebral disc space, reduced signal intensity and volume of the nucleus pulposus on T2-weighted images, asymmetric protrusion of the annulus, annular tear, and T1-weighted and T2-weighted hypointense material within the vertebral canal in continuation with the intervertebral disc [2]. Intervertebral disc extrusion was classified as thoracic if located between T1/T2 and T12/T13 and lumbar if between T13/L1 and L6/L7.

Epidural hemorrhage was identified by material within the epidural space with mixed signal intensity on T2-weighted images combined with T1-weighted iso-, hypo-, or hyperintense signals compared to a normal spinal cord and signal void on T2* GRE sequences. Extensive epidural hemorrhage was defined as the extension of epidural hemorrhage over more than two consecutive intervertebral disc spaces and expressed as times vertebral body length of L2.

Epidural material was evaluated for

- location in relation to the affected intervertebral disc space (cranial, caudal, both);
- cranio-caudal extent of the epidural material expressed as times the length of L2;
- location in relation to the spinal cord in the transverse section (dorsal right, dorsal left, ventral left, ventral right);
- location of maximal compression with respect to affected intervertebral disc space;
- maximal spinal cord compression as mild (<25%), moderate (25–50%), or severe (>50%);
- intramedullary signal void in T2* gradient echo sequences;

Recovery of deep pain perception of the DPN dogs was recorded at the time of discharge.

### Statistical Analysis

The collected variables were described by means and standard deviations when normally distributed, and median and interquartile ranges were used for non-normally distributed data. Categorical variables were expressed as numbers and percentages. Fisher’s exact test and the Chi-squared test were used for comparing the location of intervertebral disc extrusion and the proportion of EEH. NCSS was used for data analysis (NCSS 2023 Statistical Software (2023), NCSS, LLC., Kaysville, UT, USA).

## 3. Results

### 3.1. Study Population

A total of 158 French Bulldogs underwent MRI examination of the spine. Eight dogs were excluded due to loss of imaging data or incomplete sequence acquisition. Two dogs were excluded as correct counting of the vertebrae was not possible. This resulted in 148 French bulldogs being included in the image analysis, comprising 87 males and 61 females. The median age of the dogs was 4.02 years (range 0.2–8.77 years, IQR 2.15 years). The mean weight was 13.12 kg (range 7.8–18.8 kg, SD of 2.33 kg). Twelve (8.1%) had previously undergone a hemilaminectomy due to intervertebral disc disease. MRI examination was performed using a 1 T magnet for 132 French Bulldogs, a 1.5 T magnet for 4 dogs, and a 3 T magnet for 12 dogs. Of the 148 French Bulldogs 121 dogs were grouped into the DPP group (82%) and 27 were grouped into the DPN group (18%).

### 3.2. Extent of Hemorrhage and Material

Epidural hemorrhage was observed in 144 out of 148 French Bulldogs (97%). The longitudinal extent of the hemorrhage ranged from 1 to 6.5 vertebral body lengths (median 2.5). In 104 out of 148 dogs (70.3%), the epidural hemorrhage extended over more than two consecutive intervertebral disc spaces and was therefore classified as EEH. The longitudinal extent in the EEH group ranged from 2 to 6.5 vertebral body lengths (median 3); 44/148 dogs had no extensive epidural hemorrhage (non-EEH group). The extent of epidural hemorrhage in the non-EEH group ranged from 1 to 2 vertebral body lengths (median 1.5). No significant differences between groups were noted for age, sex, or body weight.

The craniocaudal extent of material in relation to the affected intervertebral disc space was seen in the cranial direction in all dogs of both groups; extension in the caudal direction was noted in all but two dogs that belonged to the non-EEH group. The median extent in the EEH group was 2× the length of L2 in the cranial direction and 1× the length of L2 in the caudal direction. For the non-EEH group, the median extent in the cranial and caudal directions was 1× the length of L2. Of the 104 dogs in the EEH group, 18 dogs (17%) were DPN; in the non-EEH group 9 of 44 dogs (20%) were DPN. There was no significant difference between both groups considering deep pain sensation (*p* = 0.6).

### 3.3. Intervertebral Disc Space

For the entire study population (148 dogs), the most frequent site of intervertebral disc extrusion was L4/L5 (33 dogs, 22%). The second most common location was L3/L4 (30 dogs, 20%), followed by T13/L1 (23 dogs, 16%) and L1/L2 (19 dogs, 13%). The affected intervertebral disc space was classified as lumbar (T13/L1–L6/L7) in 139 out of 148 French Bulldogs (94%) and thoracic (T1/T2–T12/T13) in 9 out of 148 dogs (6%). In the 104 dogs with EEH, the L4/L5 intervertebral disc space was the most affected (22 dogs, 21%), followed by L3/L4 (19 dogs, 18%), L1/L2 (16 dogs, 15%), T13/L1 (15 dogs, 14%), and L2/L3 and L5/L6 (13 dogs, 13%).

Concerning the 44 non-EEH dogs, the most commonly affected intervertebral disc spaces were L3/L4 (11 dogs, 25%) and L4/L5 (11 dogs, 25%), followed by T13/L1 (8 dogs, 18%), and less common locations were L2/L3 (5 dogs, 11%), L5/L6 (3 dogs, 7%), T12/T13 (2 dogs, 5%), and T11/T12 (1 dog, 2%).

**Figure 1:**
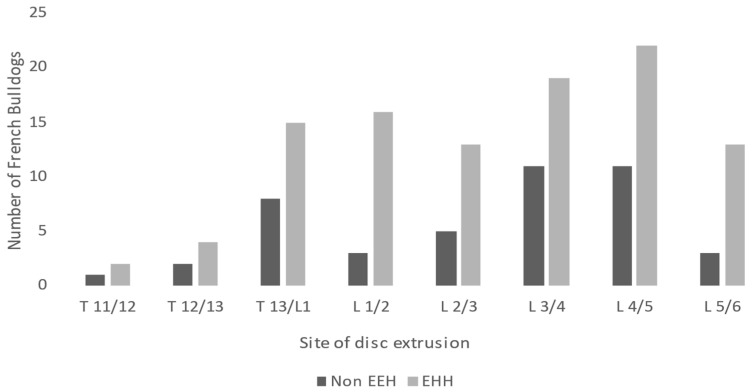
Site of thoracolumbar disc extrusion and numbers of dogs in both groups (EEH and non-EEH). EEH: extensive epidural hemorrhage; non-EEH: no extensive epidural hemorrhage.

The highest prevalence of dogs with EEH across all thoracolumbar sites of disc extrusion was seen at L1/L2 (16 out of 19 dogs, 84%), followed by L5/L6 (13 out of 16 dogs, 81%), L2/L3 (13 out of 18 dogs, 72%), and L4/L5 (22 out of 33 dogs, 66%). At the thoracolumbar junction, 15 out of 23 dogs had EEH (65%). For all lumbar sites (T13/L1–L6/L7), 70.5% exhibited EEH (98 out of 139), while, for all thoracic sites, 6 out of 9 dogs exhibited EEH (66.9%). There was no significant difference in the prevalence of EEH between the specific sites of thoracic or lumbar spine as determined by Fisher’s exact test and the Chi-square test. The numbers of dogs with EEH and no EEH for each site of disc extrusion are shown in Figure 1.

### 3.4. Involvement of Quadrants of the Vertebral Canal

**Figure 2:**
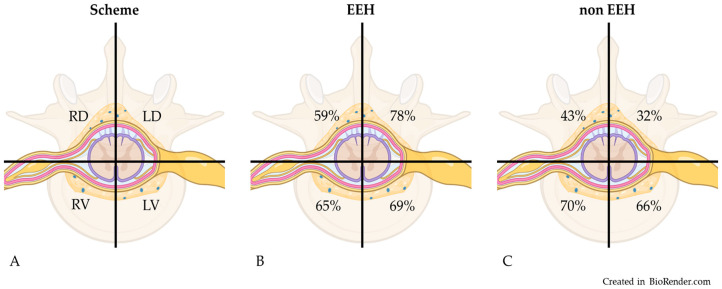
Vertebral canal as seen on transverse images divided in quadrants (**A**). For both groups, EEH (**B**) and non-EEH (**C**), the percentage of dogs with involvement of each quadrant is provided. Note that in most dogs, more than one quadrant was involved. RD: right dorsal; LD: left dorsal; LV: left ventral; RV: right ventral; EEH: extensive epidural hemorrhage; non-EEH: no extensive epidural hemorrhage.

**Figure 3:**
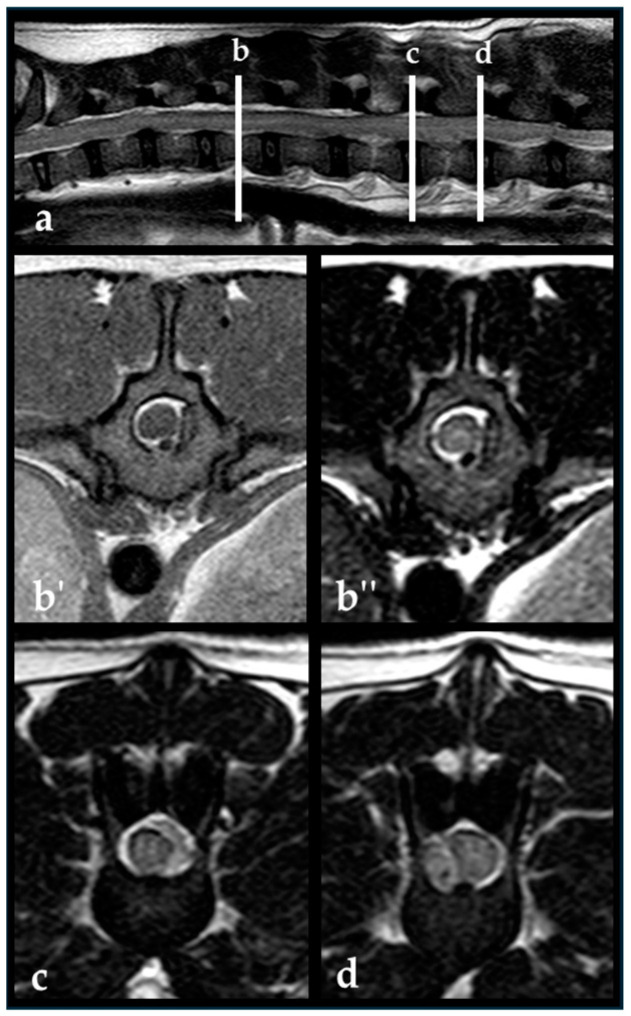
French Bulldog, 5 years, male, entire. T2-weighted sagittal image (**a**) with line b–d indicating the different levels of the transverse images. Epidural material can be seen in the right ventral epidural space at the level of intervertebral disc extrusion L3/4 (**d**). The epidural material extended cranial within the left epidural space at the level L2/3 (**c**) up to the level of mid aspect of T13 (**b′**,**b″**). The epidural material in this case expanded over 5.5× the length of L2 vertebral body lengths. The dog underwent decompressive surgery at two locations.

The evaluation of the distribution of material within the vertebral canal based on transverse images revealed that in the study group of 148 French Bulldogs, the material was in the left ventral quadrant in 101 dogs (68%), the right ventral quadrant in 99 dogs (67%), the left dorsal quadrant in 95 dogs (64%), and the right dorsal quadrant in 80 dogs (54%). The involvement of quadrants of the vertebral canal in the EEH versus non-EEH group is shown in Figure 2. Figure 3 shows a dog of the EEH group.

Material was located in only a single quadrant in four dogs: three non-EEH dogs and one EEH dog. Concerning the EEH dogs, material was present in one or both dorsal quadrants in 102/104 (98%). The involvement of one or two dorsal quadrants was seen in 30 of 44 non-EEH dogs (68%), revealing a significant difference between both groups (*p* = 0.0001). The involvement of both dorsal quadrants at the same time was observed in 40 dogs with EEH (38%) and only 4 dogs of the non-EEH group (9%). The presence of material in one or two dorsal quadrants without material in a ventral quadrant was found in four dogs with EEH (one with material in a single and three with material in both dorsal quadrants) and was not seen in dogs of the non-EEH group.

### 3.5. Compression of Spinal Cord

**Figure 4:**
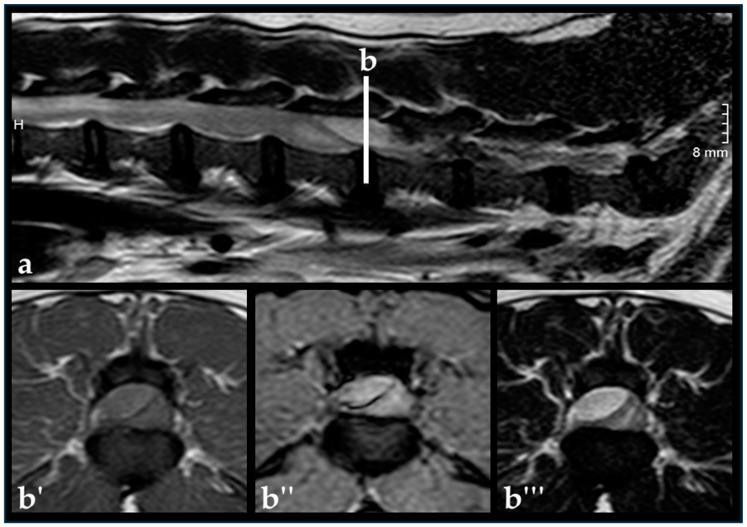
French Bulldog, 5 years, female, intact. T2-weighted sagittal image (**a**), H is indicating the direction towards the head of the dog. The white line (**b**) indicates the level of the transverse images. Transverse T1 (**b′**), T2*-weighted gradient echo (**b″**), and T2-weighted fast spin echo image (**b′′′**) at the level L4/5 showing severe compression from left dorsal due to assumed hyperacute epidural hemorrhage. The dog showed intervertebral disc extrusion at the level of L5/6. Decompressive surgery was performed at two locations.

Spinal cord compression was assessed as mild in 23 dogs (16%): 13 dogs with EEH and 10 non-EEH dogs. Moderate spinal cord compression was seen in 76 dogs (51%): 56 dogs of the EEH and 20 dogs of the non-EEH group. Severe spinal cord compression was seen in 49 dogs (33%): 35 dogs with EEH (Figure 4) and 14 dogs without EEH. There was no significant difference between both groups concerning the grade of spinal cord compression (*p* = 0.28).

The site of maximal compression was dorsal to the affected intervertebral disc space or a maximum of 0.5 vertebral body lengths away from it in 85 of 104 dogs with EEH (82%) and 41 of 44 non-EEH dogs (93%). Maximal compression at 1–2.5 vertebral body lengths away from the affected intervertebral disc space was seen in 19 of 104 dogs with EEH (18%) and 3 of 44 dogs of the non-EEH group (7%). Maximal compression of 1.5–2.5 vertebral body lengths away from the affected intervertebral disc was only seen in 7 dogs with EEH (7%). Maximal compression was at the intervertebral disc space in 18 of 44 dogs (40%) in the non-EEH group and 46 of 104 dogs (44%) in the EEH group. Maximal compression was cranial to the affected intervertebral disc space in 20 of 44 dogs (45%) in the non-EEH group and 43 of 104 dogs (41%) of the EEH group. Maximal compression was caudal to the intervertebral disc space in 6 of 44 (14%) non-EEH dogs and 15 of 104 (14%) dogs with EEH.

Lateralization of compression was seen in 99 of 104 dogs with EEH (95%) and 38 of 44 dogs of the non-EEH group (86%). Spinal cord compression without lateralization was seen in a total of 11 dogs. Six of these dogs were in the non-EEH group and showed central compression from the ventral. Five of these dogs belonged to the EEH group: four of them had circumferential compression and one dog had central compression from the dorsal.

### 3.6. Intramedullary Hemorrhage

Intramedullary hemorrhage was identified in seven dogs with EEH (6%) and one dog without EEH (2%). Two of the seven dogs of the EEH group were DPN. Both did not regain deep pain perception after surgery at the time of discharge. The remaining dogs were DPP.

### 3.7. Decompressive Surgery

Decompressive spinal surgery was performed in 134 of the 148 (90%) French Bulldogs; 14 dogs did not undergo surgery (5 non-EEH, 9 EEH). Decompressive surgery at one location was performed in 85 dogs (64%): 32 non-EEH and 53 EEH dogs. Decompressive surgery at more than one intervertebral disc space was performed in 49 dogs (36%): 42 EEH and 7 non-EEH. EEH was associated with decompressive surgery at more than one location (*p* = 0.015).

### 3.8. Recovery of Deep Pain Perception

Of the 27 DPN, 4 were euthanized before surgery and the remaining 23 underwent decompressive surgery: 18 dogs belonged to the EEH group, of which 9 did not regain deep pain perception until the time of discharge (range: 1–14 days); 5 dogs belonged to the non-EEH group, of which 4 did not regain deep pain perception. Ten dogs regained deep pain (nine EEH/one non-EEH) until the time of discharge (range: 1–10 days).

## 4. Discussion

In this retrospective study, 148 French Bulldogs with thoracolumbar intervertebral disc extrusion were assessed for extensive epidural hemorrhage (EEH). We found it especially important to include a large population of French Bulldogs because of the contradicting information in the literature regarding the association of the site of thoracolumbar intervertebral disc extrusion and EEH. A recent study of twelve French Bulldogs reported epidural hemorrhage being more commonly seen in this breed, particularly with intervertebral disc extrusion between T12 and L2 [8], while in other studies with various other dog breeds but including French Bulldogs, an association of epidural hemorrhage with a lumbar or caudal lumbar location of intervertebral disc extrusion has been reported [9]. French Bulldogs are a common dog breed in this country and are overrepresented in the hospital population. The subjective impression at our institution was that extensive epidural hemorrhage is often observed with caudal lumbar intervertebral extrusion, but the results of the present study reveal that this subjective impression may arise from the overall high incidence of lumbar intervertebral disc extrusion. In this study, the most common location of interverbal disc extrusion among all thoracolumbar sites was at L4/L5. This is of clinical interest and appears to be breed-specific as, in the general dog population, disc extrusion at the thoracolumbar junction is the most common [8,10,11,12].

In a recent study comparing dachshunds and French Bulldogs concerning malformation and disc extrusions, it was shown that intervertebral disc extrusion cranial to the thoracolumbar junction in French Bulldogs is rare, possibly because of the intercapital ligament, which may contribute to preventing intervertebral disc extrusion [13]. Concerning EEH, we found the highest incidence of EEH across all thoracolumbar sites at L1/L2 (84%), but this was closely followed by L5/L6 (82%), with little difference between the two locations. Concerning EEH at the thoracic spine, it needs to be considered that in this study group of 148 French Bulldogs, only 9 dogs had intervertebral disc extrusion in the thoracic spine, 6 of them with EEH. The small number of dogs with thoracic intervertebral disc extrusion does not allow for robust statistical analysis but demonstrates that there is only a small difference in the incidence of EEH between thoracic and lumbar intervertebral disc extrusions. Across all lumbar sites, there was no difference in incidence of EEH and no difference between the cranial and caudal lumbar spine.

The location of intervertebral disc extrusion can be an important risk factor for the development of progressive myelomalacia (PMM), especially in patients with lumbar intumescence intervertebral disc extrusions. Previous studies have shown that French Bulldogs may be at a higher risk of the development of PMM, and it could be speculated that this may be associated with the increased prevalence of caudal lumbar intervertebral disc extrusions seen in our study and others and the vascular anatomy in this area [14,15].

We found EEH in 104 dogs (70%). This number is marginally higher than expected from the reported incidences of epidural hemorrhage in French Bulldogs, which range from 41–66% [7,8].

Comparison of the incidence of EEH between different studies is difficult as not all studies specify the term epidural hemorrhage or extensive epidural hemorrhage [1,2,7,8]. We observed in almost every French Bulldog in this study some amount of epidural hemorrhage in MRI. Similar observations were made during decompressive surgery of thoracolumbar intervertebral disc extrusion in French Bulldogs, but not for cervical intervertebral disc extrusion [12]. Therefore, it seems important to differentiate between epidural hemorrhage and extensive epidural hemorrhage. In recent publications, extensive epidural hemorrhage is defined as an extension of compressive epidural hemorrhage over more than two consecutive vertebral disc spaces, which takes into account that decompressive surgery might be necessary at more than one site. As the contact area of the hemorrhage to the dura seems to play a role in triggering inflammation, it is therefore considered a possible prognostic factor [3,4]. We used the same definition as Woelfel regarding the extent of epidural hemorrhage, but, in our definition, the epidural did not necessarily need to be compressive. Applying this definition, we found a high incidence of EEH in the present study group of French Bulldogs, which is in line with previous publications, that point out the higher incidence of EEH in French Bulldogs with thoracolumbar intervertebral disc extrusion compared to other breeds. High incidences are also reported for Pitbull Terriers, Cocker Spaniels, Beagles, Labrador Retrievers, and Retriever Mixes, which have similar percentages of intervertebral disc disease with epidural hemorrhage [3,8]. Only in Dachshunds has the percentage of epidural hemorrhage been reported to be low with acute disc disease (11%) [7].

In humans, the location of spontaneous spinal epidural hematoma is described to be posterior (the equivalent to dorsal in veterinary medicine) or posterolateral within the vertebral canal, where anterior and circumferent distribution is much less common [16]. In the present study, we found tendencies of a dorsal involvement of the vertebral canal in epidural hemorrhage. In almost all dogs with EEH, at least one dorsal quadrant was involved. The involvement of both dorsal quadrants was significantly associated with EEH. Also, the finding of no involvement of ventral quadrants was only observed in the EEH group. The involvement of dorsal quadrants could help to identify epidural hemorrhage on imaging, especially if no susceptibility-weighted or CT images are available.

In most dogs, the site of maximal compression was directly dorsal to the affected intervertebral disc space. However, this showed that maximal compression cranial or caudal to the affected intervertebral disc space was more common in dogs with EEH compared to dogs without EEH. Material distributed at a distance of 1.5 to 2.5 vertebral body lengths was only observed in dogs with EEH. It remains unclear why the number of dogs with maximal compression cranial to the disc space was much higher than the number of dogs with maximal compression caudal to the affected intervertebral disc space.

In the present study, intramedullary signal voids indicating intramedullary hemorrhage were only seen in 6% of the dogs. Intramedullary GRE signal void has been associated with a significantly reduced likelihood of regaining nociception in deep pain-negative paraplegic dogs affected by intervertebral disc extrusion [17], underlining the importance of including this sequence in spinal MRI protocols for intervertebral disc extrusion [18]. Although a higher field strength should theoretically increase the free induction decay in T2*-weighted sequences and therefore improve the sensitivity for susceptibility artefacts, we noticed difficulties with T2*-weighted sequences at 3 T due to artifacts from breathing and bone and lung tissue that may limit the visibility of signal void within the vertebral canal. Recent advancements suggest that incorporating susceptibility-weighted imaging (SWI) or 2D spoiled gradient echo multi-echo sequence with magnetization transfer saturation pulse (MEDIC) into standard protocols may offer superior visualization of hemorrhagic lesions and venous structures compared to traditional T2* sequences [19,20,21].

## 5. Limitations

Limitations of the present study are the retrospective study design; records of surgical findings or histological confirmation of the extruded material were not part of the inclusion criteria. Possibly, extruded disc material might have been misdiagnosed as hemorrhage based on MRI, possibly overestimating the number of dogs with EEH. Additionally, MRI interpretation might have failed in correctly identifying the affected intervertebral disc space, leading to potentially incorrect results, which may limit the understanding of the overall disease process. Furthermore, while most MRI scans were performed using a 1.0 T scanner, three different magnets were used in this study, potentially introducing variability in imaging quality and the detection of susceptibility artifacts. The retrospective nature of this study did not allow us to examine previous corticosteroid administration and its influence on recovery, nor could a long-term outcome be analyzed for both groups.

## 6. Conclusions

L4/L5 was found to be the most common site of intervertebral disc extrusion in our large population of 148 French Bulldogs that presented with acute paraparesis/paraplegia and with an MRI diagnosis compatible with thoracolumbar intervertebral disc extrusion. A total of 70% of the dogs had imaging findings consistent with EEH. L1/L2 had the highest prevalence of EEH (84%), but no statistically significant difference was seen between the various thoracic and lumbar intervertebral disc extrusion sites and the prevalence of EEH.

## Data Availability

The raw data supporting the conclusions of this article are available from the authors upon request.

## References

[1] Tartarelli C.L., Baroni M., Borghi M. (2005). Thoracolumbar Disc Extrusion Associated with Extensive Epidural Haemorrhage: A Retrospective Study of 23 Dogs. J. Small Anim. Pract..

[2] Fenn J., Olby N.J. (2020). Classification of Intervertebral Disc Disease. Front. Vet. Sci..

[3] Woelfel C.W., Robertson J.B., Mariani C.L., Muñana K.R., Early P.J., Olby N.J. (2021). Outcomes and Prognostic Indicators in 59 Paraplegic Medium to Large Breed Dogs with Extensive Epidural Hemorrhage Secondary to Thoracolumbar Disc Extrusion. Vet. Surg..

[4] Fadda A., Oevermann A., Vandevelde M., Doherr M.G., Forterre F., Henke D. (2013). Clinical and Pathological Analysis of Epidural Inflammation in Intervertebral Disk Extrusion in Dogs. J. Vet. Intern. Med..

[5] Parker H.G., VonHoldt B.M., Quignon P., Margulies E.H., Shao S., Mosher D.S., Spady T.C., Elkahloun A., Cargill M., Jones P.G. (2009). An Expressed Fgf4 Retrogene Is Associated with Breed-Defining Chondrodysplasia in Domestic Dogs. Science.

[6] Brown E.A., Dickinson P.J., Mansour T., Sturges B.K., Aguilar M., Young A.E., Korff C., Lind J., Ettinger C.L., Varon S. (2017). FGF4 Retrogene on CFA12 Is Responsible for Chondrodystrophy and Intervertebral Disc Disease in Dogs. Proc. Natl. Acad. Sci. USA.

[7] Poli F., Calistri M., Meucci V., Di Gennaro G., Baroni M. (2022). Prevalence, Clinical Features, and Outcome of Intervertebral Disc Extrusion Associated with Extensive Epidural Hemorrhage in a Population of French Bulldogs Compared to Dachshunds. J. Vet. Med. Sci..

[8] Bridges J., Windsor R., Stewart S.D., Fuerher-Senecal L., Khanna C. (2022). Prevalence and Clinical Features of Thoracolumbar Intervertebral Disc-Associated Epidural Hemorrhage in Dogs. J. Vet. Intern. Med..

[9] Mateo I., Lorenzo V., Foradada L., Muñoz A. (2011). Clinical, Pathologic, And Magnetic Resonance Imaging Characteristics Of Canine Disc Extrusion Accompanied By Epidural Hemorrhage Or Inflammation. Vet. Radiol. Ultrasound.

[10] Macias C., Mckee W.M., May C., Innes J.F. (2002). Thoracolumbar Disc Disease in Large Dogs: A Study of 99 Cases. J. Small Anim. Pract..

[11] Aikawa T., Fujita H., Kanazono S., Shibata M., Yoshigae Y. (2012). Long-Term Neurologic Outcome of Hemilaminectomy and Disk Fenestration for Treatment of Dogs with Thoracolumbar Intervertebral Disk Herniation: 831 Cases (2000–2007). J. Am. Vet. Med. Assoc..

[12] Albertini G.M., Stabile F., Marsh O., Uriarte A. (2023). Clinical, Magnetic Resonance Imaging, Surgical Features and Comparison of Surgically Treated Intervertebral Disc Extrusion in French Bulldogs. Front. Vet. Sci..

[13] Aikawa T., Shibata M., Asano M., Hara Y., Tagawa M., Orima H. (2014). A Comparison of Thoracolumbar Intervertebral Disc Extrusion in French Bulldogs and Dachshunds and Association with Congenital Vertebral Anomalies. Vet. Surg..

[14] Olby N.J., Moore S.A., Brisson B., Fenn J., Flegel T., Kortz G., Lewis M., Tipold A. (2022). ACVIM Consensus Statement on Diagnosis and Management of Acute Canine Thoracolumbar Intervertebral Disc Extrusion. J. Vet. Intern. Med..

[15] Balducci F., Canal S., Contiero B., Bernardini M. (2017). Prevalence and Risk Factors for Presumptive Ascending/Descending Myelomalacia in Dogs after Thoracolumbar Intervertebral Disk Herniation. J. Vet. Intern. Med..

[16] Peng D., Yan M., Liu T., Yang K., Ma Y., Hu X., Ying G., Zhu Y. (2022). Prognostic Factors and Treatments Efficacy in Spontaneous Spinal Epidural Hematoma: A Multicenter Retrospective Study. Neurology.

[17] Clark R., Ferreira A., Behr S. (2023). Significance of Intramedullary T2* Signal Voids in the Magnetic Resonance Imaging of Paraplegic Deep Pain-Negative Dogs Following Intervertebral Disc Extrusion at Short-Term Follow-Up. Front. Vet. Sci..

[18] Packer R.A., Rossmeisl J.H., Kent M.S., Griffin J.F., Mazcko C., LeBlanc A.K. (2018). Consensus Recommendations on Standardized Magnetic Resonance Imaging Protocols for Multicenter Canine Brain Tumor Clinical Trials. Vet. Radiol. Ultrasound.

[19] Wang M., Dai Y., Han Y., Haacke E.M., Dai J., Shi D. (2011). Susceptibility Weighted Imaging in Detecting Hemorrhage in Acute Cervical Spinal Cord Injury. Magn. Reson. Imaging.

[20] Weston P., Morales C., Dunning M., Parry A., Carrera I. (2020). Susceptibility Weighted Imaging at 1.5 Tesla Magnetic Resonance Imaging in Dogs: Comparison with T2*-Weighted Gradient Echo Sequence and Its Clinical Indications. Vet. Radiol. Ultrasound.

[21] Held P., Dorenbeck U., Seitz J., Fründ R., Albrich H. (2003). MRI of the Abnormal Cervical Spinal Cord Using 2D Spoiled Gradient Echo Multiecho Sequence (MEDIC) with Magnetization Transfer Saturation Pulse. A T2* Weighted Feasibility Study. J. Neuroradiol..

